# Recurring Cancer Following Operation for Epithelioma

**Published:** 1873-02

**Authors:** 


### ARTICLE XI.

### *Recurring Cancer Following Operation for Epithelioma.*

In this patient, a man 40 years of age, an epithelioma of the lower lip had been removed by a triangular incision some six months before. The wound had entirely healed after the operation. A malignant growth soon began to show itself as an ulcer on the surface of the lip and on either side of the cicatrix, involving all the proper structure of the lip down to the bone. An enlarged lymphatic gland could be felt under the jaw, which had begun to take on cancerous degeneration, the cancer-cells having probably been carried there by the absorbent vessels from the seat of the disease.

Ether was administered, and the patient placed in a sitting posture to prevent the blood from running into his mouth. The surgeon then carefully removed every particle of the diseased structure, and scraped the bone, at the same time removing the enlarged gland, which he cautioned the class never to fail to do early, when it first makes its appearance, whether during the progress of the disease, or following an operation for removal of cancer of the lower lip. He invariably directs the patient, after one of these operations, in case he should find, under the lower jaw, a round tumor, like a marble, that cannot be dissipated by frictions with soap liniment, to go at once to a good surgeon and have it taken out. The way of removing it is very simple; sink a tenaculum into the gland through the skin, cut down to the gland, turn it out from the wound by pushing down the handle of the tenaculum, and pick away the gland. If the operation is delayed until the gland becomes adherent to the periosteum, it will be found too late to prevent the extension of the malignant action to the bone.

The edges of the wound were approximated by the hare-lip suture. A good result was obtained from the operation, and the wound healed shortly afterwards. The patient was warned of the liability of the disease to return, and told to apply for assistance on the first evidences of its reappearance.—*Surg. Clinic of Prof. Pancoast.—Med. Times.*

